# Paediatric fracture clinic design – current practice and implications for change

**DOI:** 10.1186/1756-0500-7-96

**Published:** 2014-02-20

**Authors:** James S Huntley

**Affiliations:** 1 Department of Orthopaedics, Royal Hospital for Sick Children, University of Glasgow, Yorkhill, Dalnair Street, Glasgow G3 8SJ, UK

**Keywords:** Fracture clinic, Paediatric, Efficiency, Torus, Buckle

## Abstract

**Background:**

In our region there has been considerable success in the redesign of adult fracture clinics. The aim of this study was to define our paediatric fracture clinic load, to assess the feasibility of increasing efficiency by decreasing inappropriate attendance.

**Findings:**

Prospective case notes review of all attendees at 6 serial fracture clinics at the Royal Hospital for Sick Children (Glasgow) which has both local and tertiary referrals. Of 234 consecutive attendances across 6 fracture clinics, 34 (15%) were judged inappropriate: 13 had fractures not requiring orthopaedic follow-up (radial torus/clavicle/undisplaced metacarpal), and 21 had diagnoses or situations that were not appropriate. Of the 200 attendances deemed appropriate (172 fractures, 11 soft-tissue injuries, 9 infections and 8 acute atraumatic limps), there were 33 new referrals from the emergency department, and a further 39 were first-time attenders at the fracture clinic after an acute admission (37 were post-operative and 2 were non-operative). Of these 200, the treatment plan was changed for 67 (34%), a cast removed or exchanged for 92 (46%), and radiographs taken for 153 (77%). The overall discharge to return ratio was 76:158 (1:2.1), and for appropriate attenders 61:139 (1:2.3).

**Conclusions:**

Tighter discipline can be applied to indications for fracture clinic appointments, including certain fracture types being discharged from the emergency department without unnecessary review - our particular fracture clinic numbers can be decreased by 15%. In the remaining attendances there are high radiograph and intervention rates, such that it seems unlikely that further reductions in attendance would be feasible.

## Findings

### Background

Fracture clinics involve the concerted action of orthopaedic surgeons, radiographers, plaster technicians and ancillary staff - in particular, secretarial and records/administration. Activity has to be co-ordinated with other services, notably theatres and the emergency department. The importance of fracture clinic organisation has long been appreciated [1,2]. In an efficient clinic, it is possible for a large case-load to be dealt with appropriately in a small time-scale [3,4].

However, some fracture clinics have high return and low discharge rates, suggesting unnecessary attendance and a need for 'redesign’ [5]. For children in particular, there is considerable associated socio-economic cost for fracture clinic attendance [6]. In our region, 'Fracture pathway redesign’ has been made 'a key priority area’ by the Scottish Orthopaedics Services Development Group [7].

The aims of this study were to define our paediatric fracture clinic load and to assess the feasibility of increasing efficiency by decreasing inappropriate attendance.

## Methods

### Fracture clinic - Royal Hospital for Sick Children, Glasgow

Yorkhill Children’s hospital in the city of Glasgow serves the population of NHS Greater Glasgow and Clyde, having a local trauma commitment in addition to a tertiary workload from the west of Scotland. There are six orthopaedic consultants who each take call, 1 week in 6, for all days of the week. Three fracture clinics are run weekly (one on each of Tuesday, Wednesday and Thursday), each under two Consultants (and these same two only and always for the particular day’s clinic) who undertake a 'buddy-system’ so that they are reciprocally available during annual/study leave periods. Usually both Consultants are present at the clinic. Attendance at a particular day’s clinic is governed by the patient being under one of the two particular Consultants. Fracture clinics are for patients originating in the acute system (emergency department or urgent referrals from primary care) and referred to the on-call Consultant with musculoskeletal conditions (predominantly fractures - but also non-traumatic limp, soft tissue injuries and infections).

Each consultant also has a separate *non-concurrent* plaster room clinic for new/follow-up patients for talipes and developmental hip dysplasia, elective casting, harness changes, and early post-operative review.

### Radiography requests

Most radiographs are requested before the clinic by the registrar or fellow who is explicitly timetabled to a session for notes and radiograph review. Sometimes it is not clear if further radiographs are indicated, in which case the patient is seen first, and only then a radiograph possibly requested.

### Data capture

All data were gathered prospectively to ensure no loss to follow-up/irretrievable notes, with real-time recording of the data prior to the release of the notes to the records department.

### Study design

This was a descriptive study over a six-week period (21/08/12-25/09/12, inclusive) in which all patients attending a Tuesday fracture clinic underwent notes review and categorisation on a predefined data sheet (not modified during study period). Referring doctors and doctors working in the clinic were not made aware of these study criteria explicitly (other than them being components of normal fracture clinic functioning) ie other than JSH, they were not aware of the study *per se*.

The data entered included:

(1) named Consultant (one of two; unless patient attending wrong clinic)

(2) diagnosis

(3) new/return to orthopaedic service; if return to orthopaedic service, were they new to fracture clinic (ie was this first fracture clinic review, after in-patient admission)

(4) appropriate attendance at fracture clinic - for reasons of clinical (including cast) review, checking fracture position or monitoring progression to union.

(5) if deemed not appropriate in question four, the reason for this – an attendance was adjudged inappropriate on the basis of any of the following four pre-defined possibilities: (a) fracture not requiring orthopaedic follow-up, (b) non acute or elective musculoskeletal condition, (c) inappropriate clinical (including cast) review, (d) wrong timescale for checking fracture position or monitoring progression to union

(6) whether or not the treatment plan was changed as a result of clinic attendance

(7) cast removed or exchanged

(8) radiograph taken at this visit

(9) patient referred to another type of health professional

(10) patient discharged.

### Statistical analysis

All information gathered in the study was recorded and analysed using the Excel software package (Microsoft, Redmond, WA, USA).

### Ethical consideration

Approval was not required as this was a simple clinical audit with no intervention, clinical or otherwise.

## Results

There were 234 attendances over the six week period. Of these, 34 (15%) were judged inappropriate for fracture clinic: 13 had fractures not requiring fracture clinic attendance (7 radius torus, 5 unilateral clavicle, 1 undisplaced metacarpal neck), and 21 were deemed inappropriate because they had non-acute or other conditions that would have been better seen in a standard elective clinic (Table 1). Four of these latter had been brought to the wrong clinic due to administrative error. Furthermore, it must be emphasized that patient attendance at the fracture clinic may be important for clinical review, without requiring a radiograph or plaster change. It is interesting that no patient attendances were classed as inappropriate by this reviewer (JSH), other than those in which the referral notes indicated either a fracture not requiring fracture clinic follow-up or a diagnosis unsuitable for management at fracture clinic.

**Table 1:** Inappropriate attendances at fracture clinic

**Reason for attendance**	***n (/21)***	**Detail**
Procedures	5	Serial casting tendo achilles (2)
		Routine ponseti casting
		Routine pavlik change (2)
Non-acute review post elective surgery	5	Wrist fusion
		Posteromedial release
		Slipped femoral epiphysis pinning
		Intramedullary wires removal
		Hip reconstruction
Long-term review non-acute conditions	4	Perthes (2)
		Hemihypertrophy + limb length discrepancy
		Developmental dysplasia hip
Acute GP rererrals	2	Neuromuscular gait disturbance
		medial clavicle swelling
Pre-operative counselling	1	Amputation for constriction band
Administrative error	4	Wrist ganglion post ultrasound
		Post botox injections review
		Longterm follow-up fibrocortical defect under different consultant
		Longterm follow-up knee septic arthritis under different consultant

The 17 patients not attributable to administrative error were all allocated to one consultant only, reflecting a heterogeneity in practice.

The remaining 200 cases (33 new and 167 return) were judged clinically appropriate for fracture clinic, for reasons of clinical (including cast) review, checking fracture position or monitoring progression to union. Of the 200 appropriate attendances, there were 172 fractures, 11 soft-tissue injuries, 9 infections and 8 acute atraumatic limps (Table 2). Upper limb injuries constituted the predominant facture-load (131/172; 76%). Thirty-three were new referrals from the emergency department (final column, Table 2), and a further 39 were first-time attenders at the fracture clinic after an acute admission (37 were post-operative and 2 were non-operative). Of the 200, the treatment plan was changed for 67 (34%), a cast removed or exchanged for 92 (46%), and radiographs taken for 153 (77%). The overall discharge to return ratio was 76:158 (1:2.1), and for appropriate attenders 61:139 (1:2.3). Eight patients (*ex* 200) were referred on to other health practitioners, of which only 3 were for physiotherapy.

**Table 2:** Appropriate attendances at fracture clinic - diagnoses

**Diagnosis**	***n (ex 200)***	**Region - *(n)***	***Region - (n)***
	**New + return**	**New + return**	**New ED referrals *(ex 33)***
**Fracture/dislocation**	**172**	**Humerus (59)**	**Humerus (6)**
		Proximal (6)	Proximal (2)
		Diaphyseal (3)	
		Supracondylar (43)	Supracondylar (4)
		Lateral condyle (7)	
		**Monteggia (5)**	
		**Coronoid (2)**	
		**Radial head (1)**	
		**Radius &/or ulna (64)**	**Radius &/or ulna (5)**
		**Femur (7)**	**Femur (1)**
		**Patella subluxation/**	
		**Dislocation (2)**	
		**Tibia &/or fibula (24)**	**Tibia &/or fibula (5)**
		**Ankle (4)**	**Ankle (1)**
		**Metatarsals (4)**	**Metatarsals (3)**
**Soft tissue injury**	**11**	**Elbow (4)**	**Elbow (2)**
		Laceration (2)	
		Sprain (2)	Sprain (2)
		**Radio-ulnar synostosis (1)**	**Radio-ulnar synostosis (1)**
		**'Clinical scaphoid’ (3)**	**'Clinical scaphoid’ (2)**
		**Wrist (1)**	**Wrist (1)**
		**Rectus femoris apophysitis (1)**	
		**Ankle (1)**	
**Acute infection**	**9**	**Abscess (1)**	
		**Cellulitis (1)**	
		**Septic arthritis (1)**	
		**Osteomyelitis (5)**	**Osteomyelitis (1)**
		Tibia (3)	Tibia (1)
		Femur (1)	
		Pelvis (1)	
		**Finger-tip crush + paronychia (1)**	**Finger-tip crush + paronychia (1)**
**Acute limp (not trauma/infection)**	**8**	**Hip (7)**	**Hip (3)**
		**Knee - sinding-larsen syndrome (1)**	**Knee - sinding-larsen syndrome (1)**

Heavy font indicates major region of body/diagnosis, with normal font indicating sub-type/diagnosis.

## Discussion

This study has been helpful in defining two groups of patients that should not be funnelled through fracture clinic: those having ongoing follow-up care for 'elective’ conditions (an unexpected finding attributable to only one of the two Consultants running the clinic) and those who could safely have been followed or discharged by the Emergency Department (ED). Once these two groups are excluded from the analysis, there was a change to treatment decision in 34%, a cast change or removal in 46%, and radiographic review in 77% ie it would appear that beyond the 'easy gains’ outlined, there is little scope for further economy, and that broadly attendances are necessary for clinical and/or radiographic review.

Our figures for inappropriate attendees referred by the ED are low in contrast to those documented for the Paediatric fracture clinic in Dublin [8], but nevertheless constitute a group that can be further reduced. Although we have identified that only a 15% reduction in clinic numbers is easily achievable, this may nevertheless contribute substantially to efficiency at the fracture clinic. This is especially likely if the inappropriate attenders from the elective side (such as 'neuromuscular gait disturbance’) consume a disproportionate amount of contact/time in this peripatetic environment.

In 2006, Morris and Bell [6] used 71 paired patient and surgeon questionnaires from 100 consecutive appointments, to examine the appropriateness and socio-economical impact of paediatric fracture clinic appointments in Sheffield, UK. From the surgeon’s questionnaire data, it was suggested that (i) the clinic appointment was appropriate in 93%, (ii) the treatment plan was changed in 25%, (iii) the cast was changed or removed in 50%, (iv) a radiograph was made in 28%, and (v) a further referral was made in 10%. Our cohort (much larger and with a complete dataset) had a similar 'appropriate’ attendee rate. It becomes awkward to make direct comparisons of other outcomes because our cohort had both inappropriate elective and fracture attendees. Taking the 200 'appropriate attendees’ as the denominator, we had a higher treatment plan alteration rate (34% *v*. 25%), a similar cast change rate (44% *v*. 50%), a dramatically higher radiography rate (77% *v*. 28%), and a lower further referral rate (4% v. 10%). Morris and Bell stated that *'The use of and adherence [to] protocols to minimise clinic attendance is essential. An understanding of the natural history of fractures in children is needed to facilitate production of protocols to allow a safe reduction in clinic appointments*’ [6]. Whilst this statement emphasises the importance of efficiency in paediatric fracture clinic design, reductions in their clinic load would rely on the assumption that the requirement for x-ray could have been made prior to assessment in the clinic [9]. It is difficult to comment on reasons why our radiography rate is higher than that encountered in the study of Morris and Bell. Our radiographs are currently requested on the same day of the clinic after previous notes and radiograph review by either registrar or fellow. One possibility is that the captured cohort differs in its epidemiology – for instance, in the proportion of cases not requiring operative intervention, being seen in other hospitals and not referred into a 'hub’ centre. In any case, our larger radiology and similar cast exchange/removal rates could be used to argue against further reductions in clinic attendance being feasible.

The current study was performed as a scoping exercise for the possibility of paediatric fracture clinic redesign. Although it contains a larger and more complete (100% case capture) data-set than the study of Morris and Bell [6], it has several limitations, notably the analysis involving the clinic of only two (of the six) paediatric orthopaedic consultants. The findings may therefore reflect particular aspects of their practice rather than that of the department as a whole. Mitigating against this, the clinical load in this clinic predominantly reflects acute on-call practice (mainly fractures), the management of which is unlikely to very substantially between Consultants. A further limitation is that data were gathered at an 'off-peak’ time of the year (autumn) - paediatric fractures are known to have particular seasonal variation [10], and therefore the findings may not reflect practice over the entire year.

A rapid review clinic of radiographs and case notes has been used elsewhere [5,11] to increase Consultant involvement/input and subsequent fracture clinic efficiency, minimising delays in later clinical decision-making. Information technology advances have certainly made such systems increasingly practicable. Whether such a system would be efficacious in our setting has not been addressed by the current study. We are fortunate in that our fracture clinics are only staffed by Consultants, clinical fellows and higher surgical registrars (peri-exam). It is possible that, in other regions, with less supervision or with more junior grades, there might be higher non-essential review or unnecessary radiography numbers.

This study has provided the data to allow our department to institute changes to our clinical practice by: (i) tighter discipline in seeing appropriate patients within the precious resource of the fracture clinic, and (ii) direct discharge of torus, undisplaced metacarpal and clavicle fractures from the ED. Although the first measure is simple, the second involves a considerable workload with protocols/education for ED doctors, as well as parent advice sheets - these requiring editing by multiple authorities.

## Competing interests

The author declares that he has no competing interests.

## Author’s contribution

JSH conceived, designed, performed and wrote up the study.
